# Economic evaluations of HBV testing and treatment strategies and applicability to low and middle-income countries

**DOI:** 10.1186/s12879-017-2778-x

**Published:** 2017-11-01

**Authors:** Shevanthi Nayagam, Elisa Sicuri, Maud Lemoine, Philippa Easterbrook, Lesong Conteh, Timothy B. Hallett, Mark Thursz

**Affiliations:** 1 Division of Digestive Diseases, Imperial College, London, UK; 2 Department of Infectious Disease Epidemiology, School of Public Health, Imperial College, London, UK; 3 Health Economics Group, Department of Infectious Disease Epidemiology, School of Public Health, Imperial College, London, UK; 4 ISGlobal, Barcelona Centre for International Health Research (CRESIB), Hospital Clinic, Universitat de Barcelona, Barcelona, Spain; 5 Global Hepatitis Programme, HIV Department, World Health Organization, Geneva, Switzerland

## Abstract

**Background:**

Many people living with chronic HBV infection remain undiagnosed until later stages of disease. Increasing testing and treatment rates form part of the strategy to respond to the WHO goal of eliminating viral hepatitis as a public health threat by 2030. However, achieving these ambitious targets is dependent on finding effective and cost-effective methods of scale up strategies. The aim of this study was to undertake a narrative review of the literature on economic evaluations of testing and treatment for HBV infection, to help inform the development of the 2017 WHO Hepatitis Testing Guidelines.

**Methods:**

We undertook a focussed literature review for economic evaluations on testing for HBV accompanied by antiviral treatment. The search was carried out in Pubmed and included only articles published after 2000 and written in English. We narratively synthesise the results and discuss the key drivers of cost-effectiveness and their applicability to low and middle-income countries (LMICs).

**Results:**

Nine published studies were included in this review, only one of which was performed in a low or middle-income setting in West Africa. Eight studies were performed in high-income settings, seven among high risk groups and one among the general population. The studies were heterogeneous in many respects including the population and testing strategy under consideration, model structure and baselines parameters, willingness to pay thresholds and outcome measures used. However, most studies found HBV testing and treatment to be cost-effective, even at low HBsAg prevalence levels.

**Conclusions:**

Currently economic evaluations of HBV testing and treatment strategies in LMICs is lacking, therefore limiting the ability to provide formal recommendations on the basis of cost-effectiveness alone. Further implementation research is needed in order to help guide national policy planning.

## Background

Hepatitis B Virus (HBV) infection is highly prevalent worldwide, with a disproportionately high burden in low and middle-income countries (LMICs) [1]. There is mounting evidence regarding the efficacy of antiviral therapy in the reduction of disease progression to cirrhosis and hepatocellular carcinoma (HCC). However, this impact is not fully translated into practise as many people still remain unaware of their infection status, in both high-income countries (HIC) [2–4] and in LMICs [5]. Wilson and Jungner criteria have been used to assess whether a disease should screened [6]. However, despite fulfilling most of these criteria [5], testing for HBV is not performed systematically. The reasons surrounding this are likely to be multifactorial, including, lack of awareness at all levels, lack of clear guidelines, competing healthcare priorities, limited healthcare budgets and political will. This leads to many people remaining undiagnosed until later stages of the disease, when prognosis is poor. Furthermore, even if diagnosed, access to appropriate antiviral therapy and ongoing clinical management is lacking.

Recognising that health care budgets are limited, economic evaluations are becoming an increasingly important tool in informing public health policy [7]. The cost-effectiveness of infant vaccination has already been established in many settings [8], antiviral treatments for HBV have been found to be cost-effective in many high-income and upper middle-income settings [9] and studies have evaluated the cost-effectiveness of various prevention of mother-to-child transmission (PMTCT) interventions for HBV; birth dose vaccination [10], birth dose vaccination combined with HBV immunoglobulin [11–13] and peripartum antiviral therapy [14, 15]. Demonstrating that testing in combination with treatment is cost-effective would provide further support for increasing testing in LMICs where economic resources are limited.

This study aimed to provide a narrative review of existing studies on economic evaluation of testing accompanied by treatment for HBV infection, to help inform the first global guidelines on viral hepatitis testing from the World Health Organization (WHO) [16]. It aims to summarise the evidence of the cost-effectiveness of testing and treatment for HBV infection, identify the key drivers of cost-effectiveness and determine how existing literature can inform optimal testing approaches that are applicable to LMICs.

## Methods

The literature was searched to identify relevant published studies on economic evaluations of HBV testing approaches and treatment. This study does not represent a full systematic review as there had been two previously published systematic reviews by Hahne et al. in 2011 [17] and Gueue et al. in 2015 [18], both looking at cost-effectiveness of screening studies in HBV and Hepatitis C Virus (HCV) infections. However, those two previous reviews concentrate on results and methodological strengths of the included studies. The aim of this study is to update and summarise the existing reviews, and draw overall conclusions about key drivers of cost-effectiveness, which would be most useful for LMIC settings. Although the burden of HBV is concentrated in LMICs, it was envisaged that there would be a lack of relevant literature in these countries, so existing studies from HICs are included and the results summarised below.

### Inclusion criteria

Studies for inclusion in this review were selected according to the following PICO framework. Population: General adult population or target populations; Intervention: Testing for chronic HBV infection accompanied by treatment (not testing prior to vaccination); Comparator: Status quo, no testing or alternative testing strategies; Outcome: Studies reporting both costs and benefits; and Study Type: Economic Evaluations (including CEAs or CBAs).

### Exclusion criteria

Studies prior to 2000 were excluded, as older studies were mainly studying cost-effectiveness of pre-vaccination testing, rather than testing for consideration of antiviral therapy. Furthermore, Geue et al., reported the low methodological standards of cost-effectiveness analyses in older studies. Non-English language articles were excluded. Studies that considered testing in blood banks, among pregnant women or healthcare workers were excluded, unless the study reported further linkage into care and treatment for the screened person. Economic evaluations which included testing for immunity prior to vaccination were excluded, unless the analysis also considered HBsAg testing and subsequent antiviral therapy for persons tested positive. Studies looking at testing for HBV prior to chemotherapy were also excluded, as this was only likely to be relevant to higher income settings and would only concern a small sub-set of the populations in LMIC. Finally, studies looking at co-infection with HIV and comparing diagnostic methods were also excluded.

### Search strategy

The bibliography of the two previous systematic reviews on the cost-effectiveness of HBV screening by Hahné et al. [17] and Gueue et al. [18] were searched and studies which met our inclusion criteria were included in our analysis. Hahne and Gueue searches were performed up to 2011 and 2012, respectively. An updated focussed search using PubMed was performed to retrieve any further relevant articles on economic evaluation of HBV testing and treatment. PubMed was searched for articles published between January 2000 and September 2016, with terms incorporating ‘Hepatitis B’, ‘HBV’, or ‘chronic hepatitis B’ and ‘Cost*’ or ‘Economic’ and ‘Screen*’, ‘Test*’ or ‘Diagn*’, in the title only. We did not search any databases other than Pubmed, nor did we search the grey literature.

Following completion of the search, relevant data was extracted using a template, which included author, year of study, country, population/setting, comparator strategies, method of economic evaluation, baseline parameters modelled (including HBsAg prevalence and cascade of care indicators) and result. The quality of each study was assessed against the Consolidated Health Economic Evaluation Reporting Standards (CHEERS) checklist [19].

#### Terminology

The term testing rather than screening is used in the WHO testing guidelines, although we use both terms interchangeably in this manuscript. We define other commonly used terms in this manuscript as follows:-.

*Cost effective:* An intervention is thought to be cost-effective when the ICER is below a given willingness-to-pay threshold (WTP). However, WTP thresholds vary between settings and are increasingly debated, particularly in LMICs [20]. A full discussion of the use of WTP thresholds is out of the scope of this current manuscript. Therefore, for the purposes of this review we used the authors’ own interpretation of cost-effectiveness and the threshold that they referred to in their respective manuscript to define a cost-effective intervention.

*High-risk groups:* For the purposes of testing for chronic HBV infection in LMIC, the categorisation of populations into ‘high-risk’ groups is not always helpful or informative in guiding policy. Currently, in many LMICs, the adult general population prevalence (unvaccinated) falls into the intermediate to high endemicity categories [21]. Furthermore, within countries, Hepatitis B surface antigen (HBsAg) prevalence is more homogenous within the population, than is the case with HCV infection. In this review, we mainly use the term ‘high-risk’ groups when referring to specific populations examined in studies from HIC settings (eg migrants, refugees). In the guidelines [16], high risk groups are those with a high risk of acquisition because of risk behaviours or exposures (to include PWIDs, MSM, HIV-infected persons).

Classification of different testing approaches: Viral hepatitis testing can be delivered to different populations and in different settings as part of general population testing, and/or a focused testing approach in most affected or high-risk populations, delivered through either health facility- based or community-based testing.

*General population testing:* This approach refers to routine testing throughout the entire population without attempting to identify high-risk behaviours or characteristics. It means that all members of the population should have potential access to the testing services. This can include community outreach testing and healthcare facility based testing.

*Focussed or targeted testing:* In the guidelines, a focussed testing approach refers to testing of specific populations who are most affected by HBV infection, either because they are part of a population with high HBV seroprevalence (such as some migrant populations and some indigenous populations), or have a high risk of acquisition because of risk behaviours and/or exposures.

## Results

### Search results

The Pubmed search retrieved 38 studies, many of which overlapped with the bibliographies of the existing reviews. After title and abstract review, finally nine published studies met inclusion criteria and are discussed in further detail in this manuscript (Table 1).

**Table 1:** Summary of studies included in the review

Author, Year	Country	Population/ Setting	Comparator strategies^a^	Data	Baseline parameters			Results^a^
					HBsAg prevalence	Cascade of Care		
						Screening uptake	Linkage to care & Adherence to treatment	
Eckman, 2011 [23]	USA	General population (primary care)	• Screen & treat (4 regimens) • No screening	CEA; Hypothetical cohort	2%	*ns*	100% adherence	$29,230/QALY *(2008)*
Nayagam, 2016 [22]	The Gambia	General population (community)	• Screen & treat • Status quo	CEA; Screening data	8.8%	68.9%	81.3% linkage, 80.9% adherence	$540/DALY ($645/LY, $511/ QALY) *(2013)*
Wong, 2011 [27]	Canada	Migrants (primary care)	• Screen & treat • Screen, treat & vaccinate • Status quo (no screening)	CEA; Hypothetical cohort	4.81%	100%	90% of all eligible will receive treatment	C$69,209/QALY *(2008)*
Rossi, 2013 [25]	Canada	Newly arrived Migrants	• Universal vac • Screen & vac • Screen & treat • Screen, treat & vac • No intervention	CEA; Hypothetical cohort	6.5%	70%	60% linkage, 75% eligible will have treatment	C$40,880/QALY *(2011)*
Hutton, 2007 [24]	USA	Asian & pacific islander adults	• Universal vac • Screen & treat • Screen, treat & ring vac • Screen, treat & vac • No intervention	CEA; Hypothetical cohort	10%	70%	100% will accept medical treatment	$36,000/QALY *(2006)*
Jazwa, 2015 [30]	USA	Overseas testing of refugees	• Vaccination only • Screen, vac & treat	CBA; Hypothetical cohort	6.8%	100%	60% linkage, 90% adherence	$90 M net benefit after 5 years^b^ *(2012)*
Veldhuijzen, 2010 [28]	Holland	Migrants	• Screen & treat • Status quo	CEA; Hypothetical cohort	3.35%	35%	58% linkage, 75% adherence	€8966/QALY
Rein, 2011 [26]	USA	Asian Migrant population (primary care & outreach)	• Comparison of 4 screening methods	CEA; Screening data	5.6–6.6%	n/a	n/a	$40- $280 per complete screen. $609–$4657 per positive case detected *(2008)*
Ruggeri, 2011 [29]	Italy	High risk groups	• Screen & treat • No screening	CEA; Hypothetical cohort	7%	100%	100% adherence	€18,225/QALY

*CEA* Cost-effectiveness Analysis, *CBA* Cost Benefit Analysis, *C$* Canadian dollar, *ns* not specified

^a^The most favourable comparator strategy is highlighted in bold and reported in the results column. The year in brackets represents the year of costs, if clearly reported in the study

^b^Value of statistical life (basecase estimated at $5 M)

### Summary of main literature

The majority of existing published economic evaluations of testing and treatment for HBV have been performed in HICs where the general population prevalence is low. Only one study was performed in a LIC setting [22]. Two studies evaluated HBV testing in the general population [23] and seven studies in ‘high-risk’ groups in HIC settings (all but one looked at testing in migrant or refugee populations) [24–30]. The studies used different methods of testing the ‘high risk groups’ including, in the clinical setting, [27, 26] community outreach methods [26] and overseas screening [30]. Eight out of 9 studies were cost-effectiveness analyses using various outcome measures including cost per QALY gained, cost per DALY averted and cost per case screened. Only one study was a cost-benefit analysis. All studies used static cohort models. Most models were simulated using hypothetical cohorts and only 2 used actual screening data to populate the model. The studies of ANC testing did not consider antiviral therapy to the mother and only looked at the benefit of testing in order to guide vaccination strategies to reduce mother-to-child transmission, and were therefore excluded in this review.

### General population level testing

The studies looking at the cost-effectiveness of offering testing and treatment to the general population were from the USA [23] and The Gambia [5].

Eckman et al., [23] examined the cost-effectiveness of HBsAg testing of asymptomatic outpatients in a primary care setting in USA to review the US guidelines on screening populations with a prevalence above 2%. They used a hypothetical cohort (35 year old male) living in a region with a prevalence of 2%. Screening was then followed by treatment with one of four regimens and compared to a no screening strategy. Screening and treatment with oral antiviral therapy was found to be cost-effective with an incremental cost effectiveness ratio (ICER) of $29,230/QALY (USD 2008). The ICER remained below their reported willingness-to-pay (WTP) threshold of $50,000/QALY gained, even down to a population prevalence of 0.3%. Limitations of this study include an unrealistic assumed 100% adherence to treatment. Furthermore, the screening costs only included the cost of a clinic visit and HBsAg testing and no sensitivity analysis was reported on the cost parameters.

The study by Nayagam et al. [22] used primary cost and effectiveness data from the Prevention of Liver Fibrosis and cancer in Africa (PROLIFICA) study [5] a large-scale intervention programme in The Gambia to determine the cost-effectiveness of adult community-based screening and treatment for HBV infection. The baseline HBsAg prevalence was 8.8%, uptake of screening 68.9%, linkage to care 81.3% and adherence to antiviral therapy 80.9%. Annual drug cost was $48, which is the generic price of tenofovir available to HIV programmes. Compared to status quo, the screen and treat strategy was found to have an ICER of $540 per DALY (USD 2013) averted ($645 per LY saved or $511 per QALY gained). The authors acknowledge that WTP thresholds levels, and their use, are highly debated in LMICs. However, it can be regarded as cost-effective if using the commonly used WHO WTP thresholds of one to three times the country’s GDP per capita to define a cost-effective intervention (GDP per capita = $487 in The Gambia [31]). This was the only published study on the cost-effectiveness of HBV screening and treatment performed in a LMIC setting. A strength of this study includes the use of primary screening data to populate the model and comprehensive sensitivity analyses on prevalence, costs and epidemiological parameters.

### Testing of ‘high-risk’ groups in HIC

There were six studies that evaluated the cost-effectiveness of screening and treatment in migrant or refugee populations in HICs [24–28, 30], and one examined screening all groups classified as ‘high risk’, in accordance with Italian guidelines [29].

The study by Wong and colleagues in 2011, looked at the cost-effectiveness of screening and treatment of immigrants for CHB, in Canada [27]. They considered a screen and treat strategy and a screen, treat or vaccinate strategy, with status quo (no screening). Screening was offered by the primary care physician at a visit scheduled for another reason, described by the authors as a ‘case-finding’ strategy. They used a hypothetical cohort (35 year old male) with a baseline HBsAg prevalence among the immigrant population of 4.81%. Screening uptake was 100% and it was assumed that 90% of those eligible would receive treatment. The screen and treat strategy (with tenofovir) had an ICER of C$69,000/QALY gained (Canadian$ in 2008). The authors acknowledge the uncertainty around WTP thresholds and describe this is as likely to be a moderately cost-effective intervention. The study explores cost-effectiveness by country of origin, revealing higher cost-effectiveness of a screen and treat strategy for immigrants born in east Asia, central and West Africa, corresponding with higher prevalence rates. A strength of this model is that it is more clinically representative of HBV than many of the other models. However, it uses high, and probably unrealistic, assumptions for uptake of screening.

Another Canadian study by Rossi et al. (2013) [25] looked at combinations of scenarios involving screening, treatment and vaccination, among newly arrived immigrants and refugees. A hypothetical cohort of 250,000 immigrants was modelled using a societal perspective. The baseline assumptions were 70% acceptance of screening, 60% linkage to care, 75% of those eligible will have treatment and annual cost of antiviral drugs $8089. The screen and treat scenario was found to be the most cost-effective with an ICER of C$40,880/QALY gained (Canadian $ in 2011) and remained robust over all one way sensitivity analyses. This strategy exceeds the Canadian WTP threshold adopted in this study of  C$50,000/QALY, when HBsAg prevalence is less than 3%.

An earlier study by Hutton et al. [24] looked at the cost-effectiveness of screening and vaccination of Asian Pacific Islander adults for HBV, comparing four strategies of combinations of screen, treatment and vaccination. They adopted a societal perspective and used a hypothetical cohort with an average age of 40 years and a HBsAg prevalence of 10%. The screen and treat strategy was the most cost-effective with an ICER of $36,000/QALY (USD in 2006) gained (compared to no screening), even down to an HBsAg prevalence of 1%.

Another, more recent, US study by Jezwa and colleagues [30], compared the cost-benefits of two overseas programmes for reducing HBV infection among refugees. They compared two strategies i. vaccination only and ii. screening, vaccination and suggested onward treatment on arrival in USA if HBsAg positive. Their baseline assumptions included a HBsAg prevalence of 6.8%, 100% adherence with screening, 60% of those tested positive for HBsAg linked to specialist care and 90% adherence to treatment. This was the only economic evaluation which adopted a cost-benefit method, where mortality risk reduction benefits were estimated using a value of statistical life approach (VSL). They found that the screening strategy had a positive net benefit of $90 million after 5 years, when VSL was estimated at $5 million (USD 2012). A strength of this study was the use of original data sets of refugee populations in two US states for the epidemiological data.

The study by Veldhuijzen et al. [28] was the only European study which looked at the cost-effectiveness of HBV screening & early treatment of migrants. An active screening method was used, where the target population is identified using the municipal population registry and they receive a postal invitation to attend screening. Their baseline HBsAg prevalence was 3.35%, with 35% participation rate in screening, 58% linkage to specialist care and 75% adherence. Compared to status quo, screening and treatment had an ICER of €8966/QALY gained and was therefore reported as cost-effective compared to the authors’ reported WTP threshold of €20,000/QALY gained. This study found that despite using low rates of participation throughout the cascade of care, that screening is still likely to be cost-effective. A strength of this study was the inclusion of comprehensive screening programme costs including personnel costs.

A study by Rein et al. [26] looked at different methods of screening for HBV among the Asian migrant population in the USA, using actual screening data. This was a descriptive study with outcome measures given as cost per person screened and cost per positive case detected. The screening methods analysed included testing at a community clinic and other more active community outreach models where screening was performed at various events in the Asian community. The costs per person screened ranged from $40 to $280 depending on the method used. Integrating screening into clinical services was found to be the least costly method, but reached least people, whereas extending screening outside the clinical setting was more costly as it included costs of organising events and volunteer time, but reached more people. This study provides useful insights into the relative costs of various screening methods and, unlike some of the other studies, it includes full costs including those associated with recruiting patients. However, it does not provide long term outcomes following on from a positive screening test and is therefore limited in its generalizability.

Ruggeri et al. [29] looked at screening of all groups defined as ‘high risk’ (according to local Italian guidelines), and compared the cost effectiveness of screening followed by antiviral treatment for CHB. This was compared to the status quo strategy of no screening, but treatment for cirrhosis and HCC stages only. A hypothetical cohort of 100,000 individuals was considered and screening and treatment was found to be cost-effective with an ICER €18,255/QALY. However, this study included treatment with suboptimal drug combinations and was based on unrealistic 100% adherence rates to testing and treatment.

## Discussion

Overall, the studies found that testing and treatment for chronic HBV infection was likely to be cost-effective among high prevalence groups (such as migrants or refugees) in HICs and among the general population in a LIC with high general population seroprevalence. The findings were relatively robust over a wide range of parameters tested. However, the studies were highly heterogenous in terms of their settings, model structures, assumptions and willingness-to-pay thresholds used. This review highlighted the lack of existing literature on the cost-effectiveness of testing and treatment in LMICs. The existing literature included only two previous community-based cost-effectiveness of testing studies, and further studies of testing among groups classified as ‘high-risk’ including immigrant populations. Most studies modelled cost-effectiveness, with only a few estimating cost-effectiveness directly from clinical studies and assumed high rates for adherence to the care cascade.

WHO GDG recommendations were formulated based on consideration of evidence from cost–effectiveness analyses together with data on HBsAg seroprevalence in different settings and populations, with considerations of overall balance of benefits and harms, feasibility and cost. The caveats of extrapolating cost-effectiveness data from HICs to LMICs were recognized. Overall, given the heterogeneity and limited number of studies the evidence-base used to inform recommendations to the WHO Clinical Guidelines Committee was rated as low quality, based on the GRADE system [32]. The WHO Testing Guidelines recommend general population (conditional recommendation) and ANC testing of pregnant women (strong recommendation) in settings where the HBsAg prevalence is >2% and focused testing in populations classified as high risk in all settings (strong recommendation) [21]. Although general population testing was estimated to be cost–effective down to prevalence levels <1%, the GDG proposed a higher threshold of ≥2% to reflect the well accepted thresholds for defining intermediate (≥2%)/high (≥5%) seroprevalence. It was recognised that the threshold used by countries will depend on other country considerations and epidemiological context.

### Drivers of cost-effectiveness

Several key drivers of cost-effectiveness were identified in the review, and are summarized below. Although the results of the studies performed in HICs are not directly comparable quantitatively to those performed in LMICs, in the absence of other literature on testing and treatment for HBV, we have extrapolated and drawn comparisons, where appropriate.

#### Costs

Economic evaluations of testing and treatment for HBV should ideally include costs of screening, diagnostics, monitoring and drugs, taking into account, both the cost of consumables as well as the cost of delivering the intervention (eg human resources), although the latter is often captured poorly in studies. The variability in all these cost components across different settings and over time, makes comparison of studies difficult. Screening costs varied between the studies; in the USA the Rein [33] study reported costs per person screened between $40 to $280, with the higher costs representing the more active outreach strategies and in The Gambia, community-based screening costs were low ($7.43 per person offered screening). Screening costs were only found to be drivers of cost-effectiveness in the Wong [27] and PROLIFICA studies. However, in the Gambia, the intervention remained below a 3 times GDP per capita WTP threshold, even if there was a 3-fold increase in screening costs.

A key driver of the cost-effectiveness of a screen and treat strategy reported in some studies is the cost of the antiviral drug [22, 24, 25]. The Rossi study used a baseline drug cost of $8089/year, to represent the average cost of tenofovir and entecavir. They varied this between $7000–$9100/year in their sensitivity analysis and this changed the ICER by $10,000, while still remaining below their reported WTP threshold. In the PROLIFICA study, the generic price of tenofovir ($48) available for use in HIV programmes in SSA [34] was used as the base-case. Using the 2014 pharmaceutical drug price of $207 [35] decreases the cost-effectiveness of the screening strategy, as it increases the ICER to $1064/DALY averted.

The cost-effectiveness of screening and treatment strategies in HIC settings is attributable in part to the fact that early management reduces the risk of long-term sequelae, which can incur significant costs (for example, estimated costs of managing cirrhosis and HCC is $9000 and $15,000 per person per year, respectively, in the Canadian study by Rossi et al. [25]). However, in LICs, there are currently limited options for management of end-stage liver disease (for example, no transplant services, limited endoscopy facilities and limited palliative care) and patients often die at home, with family as primary care-giver. Therefore, more studies are needed to evaluate whether the costs of the intervention, from a healthcare perspective, offset the cost avoided of end stage liver disease. A societal perspective analysis might be more appropriate for future cost-effectiveness studies in LMICs. Furthermore, the annual costs of managing liver disease are variable and largely unknown and should be the subject of further research [36].

#### HBsAg prevalence

Although the studies varied in the baseline HBsAg prevalence used in the model (2–10%), they reported how the cost-effectiveness of the intervention would change over a range of HBsAg prevalence levels. HBsAg prevalence was found to have a relatively small influence on cost-effectiveness, over the wide ranges evaluated, in most of the studies. General population screening was found to remain cost-effective (ie ICER below the respective WTH threshold) down to a HBsAg prevalence of 0.3% in the USA [23] and 1.5% in The Gambia [22]. Screening of migrants in North America remained cost-effective down to prevalence levels of 1% [24] - 3% [37]. An important caveat is that there is inter-study variation as to how ‘cost-effectiveness’ is assessed; each using differing scales of cost and WTP thresholds - concepts that are also debated [38]. Therefore extrapolation of the HIC results to LMIC is difficult, and the absolute threshold cut-off for HBsAg prevalence cannot be guided based on the existing literature alone. However, there is concordance between studies of cost-effectiveness of such a strategy even at low HBsAg prevalence levels. This has important implications for choice of testing approach when considering testing in other countries with different prevalence profiles and different levels of historical vaccination coverage.

#### Patient Behaviours

Adherence to treatment and linkage to care were reported as key drivers of cost-effectiveness in some of the studies [25, 28]. Veldhuijzen et al. reported that variation in rates of linkage to care and treatment adherence had the largest influence on ICER (ICER varied by about €3000 over the ranges tested, 39–75%, 50–100%, for linkage and adherence, respectively). In contrast, in The Gambia, these factors were less influential on the cost-effectiveness. Uptake of screening is not reported to be a key driver of ICER in the studies.

#### Disease progression rates

Although the HBV economic evaluations varied in their natural history models and baseline parameter assumptions, most showed that the cost-effectiveness was relatively sensitive to variations in disease progression rates. The Dutch study [28], showed that varying parameters between a range representing fast disease to slower disease progression, showed significant variation in ICER between €5000 and €60,000/QALY gained, respectively, a trend which was also seen in other studies [24, 27]. The Eckman study showed that the ICER was most sensitive to the rate of spontaneous HBeAg seroconversion assumed to be 5% at baseline, but exceeded the WTP threshold if increased to 10%. The study in The Gambia also showed that many of the transition rates, were influential on ICER [22]. However, given the complex and heterogenous natural history of HBV, both within and between populations, and lack of natural history progression rate data specific to all populations, this is likely to remain a limitation of all HBV models.

#### Effectiveness of antiviral therapy

Effectiveness of antiviral therapy was found to be influential on ICER in some studies [24, 27], but not in others [22]. This discrepancy is likely to be due to study differences on the type of antiviral drug used and efficacy assumptions (some of which have been superseded with more current data). For example, the older studies often included low-barrier to resistance drugs like lamivudine or interferon, whilst the newer studies mainly used tenofovir or entecavir. Therefore, conclusions as to the influence of these parameters on the result, as well as comparisons between studies have to be interpreted with caution.

#### Other factors

The assumptions which underlie the methodological approach taken will also affect the conclusion of cost-effectiveness studies, for example, health utility values used for Quality Adjusted Life Year (QALY) assumptions [23, 27] discount rate [27, 30] and the cost-effectiveness thresholds used [39]. Although guidelines exist as reference for the techniques used, the variability between studies limits accurate comparisons. A full discussion of these factors is outside the scope of this current manuscript.

### Generalisability of results

There are several key challenges in the use of these cost-effectiveness studies to inform WHO recommendations on testing approaches. WHO recommendations are primarily to guide strategy decisions in LMICs, but the literature for HBV in these countries is sparse. Direct comparison of results from cost-effectiveness analyses between countries or regions with such differing health care structures, costs, patient behaviors, disease prevalence profiles and willingness-to-pay thresholds can be misleading and needs to be interpreted with caution. However, funding, timescales and resources are unlikely to be available for individual country-level analyses to be a realistic goal across disease areas, before a new testing strategy is implemented. Therefore, some cautious inference can be drawn from existing literature. Other limitations of this review include the fact that the search was only conducted in Pubmed and limited to English language articles only, therefore excluding further potentially relevant studies particularly from LMICs. The heterogeneity in methods, settings and assumptions used between models also limit the quantitative synthesis of study results.

### Considerations regarding where to test

Careful consideration needs to be given regarding alternative places to increase testing opportunities for HBV infection, whilst ensuring linkage to care for treatment. Community-level testing may be considered the most active type of case-finding approach with outreach components and therefore likely the most labour and resource intensive. However, within PROLIFICA, it was found to be cost-effective, with low testing costs of $7.43 per person offered testing. Various examples of community outreach programmes exist in the field of HIV [40], and comparable strategies could be considered for HBV, with the caveat that ‘high risk’ groups will not be as applicable to HBV infection. However, a strategy of active door-to-door testing may not be a feasible in all settings. The use of media campaigns to sensitise and educate the population about the benefits of early testing for HBV could be an alternative method to increase testing, and there is a need for further implementation research to evaluated the impact of such campaigns in LMICs. Health-care facility testing includes primary care, inpatient and outpatient settings or specialist clinics (eg HIV or STD clinics), and can be used for both routine general population testing approaches as well as those with a clinical indication for testing (eg abnormal liver function tests, abnormal ultrasound scan, family history of liver disease or other clinical suspicion of liver disease). A clinically guided testing approach is likely to reveal a higher proportion of people with HBV in highly endemic settings and could be cheaper, but would be limited in its reach and ability to pick up early asymptomatic infection, and would need to take into account the mortality of the sub-population seeking healthcare.

The screening of pregnant women for HBsAg (with or without HBeAg testing) in antenatal care (ANC) settings has been considered in previous cost-effectiveness analyses, as was not a focus of this current narrative review. A limitation of these previous studies was that they only considered the reduction in MTCT and benefits to the child. None considered onward linkage into care or treatment for the mother, to reduce their risk of progression of liver disease. Furthermore, many were older studies published before 2000 (summarised in a review by Hahne et al. [17]) and were performed mainly in HICs. Cost-effectiveness analyses in this setting needs to balance the lower incremental costs of HBV testing during an already planned ANC visit, with the lower HBsAg prevalence and slower rates of progression to HCC among women. However, since testing of mothers for HBV has benefits to both the mother and child, this is likely to be cost-effective. Since there is variable percentage of attendance to antenatal care depending on geographic region [41] this approach should also consider factors which will help strengthen ANC coverage overall, including increasing patient education.

Blood donor screening for HBV already forms part of WHO recommendations in order to prevent transmission of blood-borne viruses to the recipient [42]. However, in LMICs, this is rarely accompanied by the HBsAg positive donor being informed of this positive result, counselled and linked into care for clinical evaluation and treatment [43]. As part of the PROLIFICA study in The Gambia, blood donors who had tested HBsAg positive at the blood bank were linked into care and offered antiviral therapy [5]. The cohort of blood donors, compared to those who were screened in the community, had a higher proportion who tested HBsAg positive, majority males, younger ages, a higher proportion requiring treatment and a lower proportion who linked to care. However, as blood donors form only a small fraction of the population, this approach is likely to be limited in its reach and population level effectiveness and probably should be seen as a complementary, rather than alternative to a wider screening strategy.

Although other methods of testing are used, to varying levels, worldwide, including testing in work places, testing of healthcare workers, couples pre-marriage, military recruits or pre-employment screening, implementation and guidance of these methods are highly heterogenous between countries [48] and are not accompanied by effectiveness or cost-effectiveness data. Care must also be taken to avoid testing methods which are associated with stigma or discrimination.

## Conclusions

This review has highlighted the paucity of data on the cost-effectiveness of testing and treatment for HBV infection, especially in LMICs. However, although it was not possible to formulate strong recommendations regarding the best testing approach, based on cost-effectiveness data alone, overall our narrative review of available studies suggests that offering testing to the general population with subsequent antiviral treatment is cost-effective in HICs [23], as well as LICs [22], even when the prevalence is low. Furthermore, testing also has benefits, which extend beyond the person tested, for example prevention-of-mother to child transmission. Ultimately, each country must determine its optimal testing approach based on local epidemiology, healthcare infrastructure and resources. The 2017 WHO testing guidelines includes a strategic framework to guide countries’ decision-making on selecting testing approaches. The WHO guidelines recommend that testing approaches in the general population and focussed testing in most affected/higher-risk populations should make use of existing community-based or health facility testing opportunities or programmes such as at antenatal, HIV or TB clinics. A further emphasis was on ensuring linkage to prevention, care and treatment services following testing.

Further large-scale operational research is urgently needed in other high-endemic, low-income settings. Furthermore, in addition to cost-effectiveness analyses, economic evaluations that examine the affordability and budgetary impact of scaling up testing and treatment interventions for HBV would be helpful in policy planning. Improving country access to generic priced tenfovir for HBV mono-infection in all LMICs is also vital to allowing adoption of affordable widescale HBV treatment programmes, which is still not the case in most of SSA, outside the remit of the HIV programme. Furthermore, HBV screening costs could be shared across other disease programmes, as there are overlapping benefits and synergies with maternal and child health goals and HIV infrastructure and experience.
